# The Relationship Between Serum Bilirubin Concentration and Atrial Fibrillation

**DOI:** 10.4021/cr299w

**Published:** 2014-01-02

**Authors:** Mehmet Demir, Canan Demir, Umut Uyan, Mehmet Melek

**Affiliations:** a Cardiology Department, Bursa Yuksek Ihtisas Education and Research Hospital, Bursa, Turkey; b Infectious Disease Department, Bursa Sevket Yilmaz Education and Research Hospital, Bursa, Turkey

**Keywords:** Arrhytmia, Atrial fibrillation, Bilirubin, Inflammation, Endotel dysfunction

## Abstract

**Background:**

Several studies have demonstrated that higher serum bilirubin inhibits the inflammation and proliferation of vascular smooth muscle cells; also there is a relationship between serum bilirubin and cardiovascular disease. However, the relationship between bilirubin and atrial fibrillation (AF) is still unknown. In our study, we compared serum bilirubin, between nonvalvular AF patients and controls.

**Materials and Method:**

One hundred and two patients with nonvalvular chronic AF without any other cardiovascular disease (mean age 62.51 ± 5.88) were included in our study. One hundred age-matched healthy people with sinus rhythm were accepted as control groups (mean age 61.35 ± 5.44). Routine biochemical parameters and serum bilirubin levels were performed.

**Results:**

No statistically significant difference was found between two groups in terms of basic characteristics. Total, direct and indirect serum bilirubin levels were significantly lower among persons with AF compared to controls (0.82 ± 0.8 vs. 0.48 ± 0.5, 0.30 ± 0.2 vs. 0.19 ± 0.1 and 0.52 ± 0.5 vs. 0.29 ± 0.3 mg/dL; all P < 0.001, respectively).

**Conclusion:**

As a result, our study revealed a relationship between serum bilirubin and nonvalvular AF.

## Introduction

Atrial fibrillation (AF) is the most common cardiac rhythm disorder. The estimated prevalence of AF ranges from 0.4% to 1% in the general population and increases by age. AF is an important public health issue, which increases the risk of mortality, stroke and cardiac failure [1-3].

Several previously published studies have demonstrated the relationship between serum bilirubin levels and cardiovascular disease such as coronary artery disease. Bilirubin is an important and potent endogen anti-oxidant and anti-inflammatuar [4-6].

Elevated levels of systemic inflammatory markers have been found associated with incidence of cardiovascular diseases such as coronary artery disease, hypertension and AF [7-9]. It is also known that inflammation plays an important role in initiating AF and in electrophysiologic remodelling.

As far as we know, there is no study performed until today about the association of serum bilirubin with AF. In our study, we compared total serum bilirubin levels, between nonvalvular AF patients and controls.

## Materials and Methods

The study included 102 patients with nonvalvular chronic AF without any other cardiovascular disease (42 male; average age 62.51 ± 5.88). One hundred age-matched healthy people with sinus rhythm were accepted as control groups (40 male; mean age 61.35 ± 5.44).

Exclusion criteria included the presence of the following: known coronary artery disease, chronic renal failure, chronic liver disorders, moderate or severe valvular disease, diabetes mellitus, hypertension, congenital heart disease, left ventricular (LV) systolic dysfunction on echocardiography (ejection fraction (EF) < 50%), recent acute coronary syndrome, anemia, hyperthyroidism, pregnancy, obstructive sleep apnea, secondary hypertension, hematologic disorders, known malignancy and drug history including anti-gout agent, WBC count > 12,000 cells/µL or < 4,000 cells/µL and high body temperature > 38 °C. Also patients that had a recent history of an acute infection or an inflammatory disease are excluded from the study. Written informed consent was obtained from all the patients.

The blood pressure of the patients was measured. The patients having a systolic blood pressure ≥ 140 mmHg and/or a diastolic blood pressure ≥ 90 mmHg and those taking anti-hypertensive drugs were accepted to be hypertensive. The patients using oral anti-diabetic drugs or insulin or those having two measurements of fasting blood glucose level ≥ 126 mg/dL were accepted to be diabetic.

### Laboratory analyses

Fasting blood glucose, serum bilirubins, creatinine, total cholesterol, electrolits and thyroid-stimulating hormone levels were recorded. Blood samples were drawn by venipuncture to perform routine blood chemistry.

### Echocardiographic measurements

Two-dimensional, M-mode, pulsed and color flow Doppler echocardioagraphic examinations of all subjects were performed by the same examiner with a commercially available machine (Vivid 7 pro, GE, Horten, Norway, 2 - 4 mHz phased array transducer). During echocardiography, a one-lead electrocardiogram was recorded continuously.

M-mode measurements were performed according to the criteria of American Society of Echocardiography [10, 11]. Left atrium (LA) diameter, LV end-systolic and end-diastolic diameters were measured. LVEF was estimated by Simpson’s rule.

### Statistical analysis

SPSS 16.0 statistical program (SPSS Inc., Chicago, IL, USA) was used for statistical study. All values are given as mean ± standard deviation. Mean values of continuous variables were compared between groups using the Student’s t test or Mann-Whitney U test, according to whether normally distributed or not, as tested by the Kolmogorov-Smirnov test. In order to define the relationship between AF and possible confounding factors, logistic regression analysis was used. A P value of less than 0.05 was considered significant.

## Results

Evaluating basic clinical and demographic characteristics, there was no statistically significant difference between two groups in terms of age, gender distribution, body mass index and smoking status (Table 1).

**Table 1:** Comparison of Basal Demographic, Biochemical and Hemathological Features of AF Patients and Controls

	AF (n = 102)	Control (n = 100)	P value
Age (years)	62.51 ± 5.81	61.35 ± 5.44	NS
Sex (males) (n, %)	42 (41.2%)	40 (40%)	NS
BMI (kg/m^2^)	22.96 ± 3.35	23.49 ± 4.39	NS
Smoking (n, %)	22 (21%)	19 (19%)	NS
Fasting glucose (mg/dL)	97.7 ± 15.1	95.2 ± 11	NS
Creatinin (mg/dL)	0.88 ± 0.9	0.75 ± 0.19	NS
AST (U/L)	24.2 ± 4.4	23.9 ± 3.6	NS
ALT (U/L)	21.1 ± 2.5	21.0 ± 2.4	NS
Total cholesterol (mg/dL)	188.5 ± 36	199.6 ± 21	NS
Na (mmol/L)	140.1 ± 17	139 ± 15	NS
K (mmol/L)	4.2 ± 0.4	4.3 ± 0.8	NS
Total bilirubin	0.48 ± 0.5	0.82 ± 0.8	< 0.001
Direct bilirubin	0.19 ± 0.1	0.30 ± 0.2	< 0.001
Indirect bilirubin	0.29 ± 0.3	0.52 ± 0.5	< 0.001
TSH (µIU/mL)	1.50 ± 1.3	1.62 ± 1.3	NS
Hemoglobin (g/dL)	14.1 ± 1.4	14.0 ± 1.4	NS
Platelets (10^3^/mm^3^)	233 ± 68	242 ± 75	NS
Leukocyte (10^3^/mm^3^)	6,811 ± 125	7,925 ± 263	NS

BMI: body mass index; NS: nonsignificant; AST: aspartate aminotransferase; ALT: alanine aminotransferase; TSH: thyroid-stimulating hormone.

Serum total bilirubin, direct bilirubin and indirect bilirubin were lower in patients with AF than controls (0.82 ± 0.8 vs. 0.48 ± 0.5, 0.30 ± 0.2 vs. 0.19 ± 0.1 and 0.52 ± 0.5 vs. 0.29 ± 0.3 mg/dL; all P < 0.001, respectively). Other hematologic and biochemical parameters were not statistically significantly different in among the two groups (Table 1).

When the initial conventional echocardiographic parameters of the patients were evaluated, the average LA diameter of the group of the patients with AF was significantly larger than that of the control group individuals (P < 0.001). The average systolic pulmonary artery pressure of the group of the patients with AF was significantly higher than that of the control group individuals (P < 0.001) (Table 2).

**Table 2:** Comparison of Echocardiographical Features of AF Patients and Controls

	AF (n = 102)	Control (n = 100)	P value
LV EF (%)	63.14 ± 4.65	63.41 ± 4.77	NS
LV ESD (mm)	28.27 ± 3.18	27.8 ± 3.54	NS
LV EDD (mm)	46.78 ± 3.11	46.27 ± 3.75	NS
IVSd (mm)	10.32 ± 0.97	10.24 ± 1.34	NS
PWd (mm)	9.41 ± 0.96	9.31 ± 1.2	NS
LAd (mm)	44.1 ± 2.15	37.44 ± 3.54	< 0.001
RAd (mm)	32.65 ± 3.99	31.9 ± 3.14	NS
PASP (mmHg)	33.17 ± 3.45	29.92 ± 3.38	< 0.001

LV: left ventricular; EF: ejection fraction; EDD: end-diastolic diameter; ESD: end-systolic diameter; IVSd: interventricular septum diameter; PWd: posterior wall diameter; LAd: left atrium diameter; RAd: right atrium diameter; PASP: pulmonary artery systolic pressure.

There was not statistically significant difference between the groups in terms of the average end-diastolic and end-systolic LV diameter, LVEF, thicknesses of interventricular septum and posterior wall (Table 2).

To determine the independent indicators of AF, a forward stepwise logistic regression model was established. In this model, AF was inserted as the dependent variable while gender, age, body mass index, the diameter of the LA, the diameter of the right atrium, the LVEF and total serum bilirubin level were entered as independent variables. As a result of the forward stepwise logistic regression analysis, it was found that LA diameter and serum total bilirubin level were the independent predictors of AF (Table 3).

**Table 3:** Logistic Regression Analysis

	HR (95% CI)	P
Left atrium diameter	2.29 (1.810-2.920)	< 0.001
Serum bilirubin	0.82 (0.780-0.950)	< 0.001

## Discussion

In the present study, we have found that total serum bilirubin levels are significantly lower in AF groups compared to controls.

AF is a commonly encountered arrhythmia, which reduces the quality of life of the patients and negatively affects the prognoses of the present diseases. Therefore, impeding the development of AF and protective treatments become more popular today.

Epidemiologic studies have demonstrated that cardiac failure, valvular heart diseases, hypertension and ischemic heart disease are the most common causes of AF [2]. Several mechanisms have been proposed for AF development. Several of these mechanisms are the atrial wall tension, inflammation and fibrosis.

The atrial biopsy specimens of lone AF patients revealed inflammatory cell infiltration and fibrosis signs. Oxidative stress and inflammation plays an important role in atrial remodelling. The relationship between the inflammation and AF was researched using CRP levels. CRP levels were found to be significantly high in the situations in which AF is common. It is thought that CRP plays a role in the complement-mediated inflammation [12]. Frustaci et al [13] evaluated the atrial biopsies of AF patients and found inflammatory infiltration, myocyte necrosis and fibrosis. Similar findings were found in animal models developed by Nakamura et al; active atrial peri-myocarditis, inflammatory infiltration, lipid degeneration and fibrosis were found in the dogs with AF [14]. In a retrospective, case-control study conducted by Chung et al with 131 AF patients and 71 healthy individuals, it was found that AF was associated with doubled CRP levels. In that study, independent predictors of AF were found to be age, HT, DKMP, female gender and less powerfully high CRP levels [15].

That the statins have anti-inflammatory effects and presumed association of AF with inflammation has accelerated the researches about their effects on AF. Siu et al, in a retrospective study with lone AF patients, showed that AF recurrences were lower among the patients given simvastatin or atorvastatin treatment before cardioversion in comparison with the patients who did not receive lipid-lowering medications (42% vs. 84%, P = 0.032) [16].

The relationship between low bilirubin levels and increased cardiovascular risk is well known.

However, there are no studies on specific relationships between the inflammatory cells and AF.

Recently, low serum bilirubin level has been proposed as a useful biomarker to predict cardiovascular risk. Recent evidence suggests that bilirubin acts as a potent physiologic anti-oxidant and anti-inflamatuar. Recently studies have shown that elevated serum bilirubin concentrations provide important protection against atherosclerotic diseases [17-19].

Previous studies have shown that plasma bilirubin concentrations are correlated inversely with several risk factors for coronary artery disease such as smoking, diabetes and obesity, and correlated directly with HDL cholesterol [20, 21].

The inverse correlation between the presence of coronary artery disease, peripheral arterial disease, carotid intima-media thickness and bilirubin was reported in several studies. Subnormal levels of plasma bilirubin are associated with premature coronary artery disease and cardiovascular morbidity [22, 23].

In a previous study, the 3-year incidence of coronary artery disease was significantly lower in patients with Gilbert syndrome [24].

Elevated concentrations of plasma bilirubin were suggested to be able to prevent atherogenesis. Strong ability to scavenge peroxyl radicals and the anti-oxidant capacity of bilirubin functioning even in slightly increased concentration in circulation have led to the concept that it may have a physiologic function to protect against disease processes involving oxygen and peroxyl radicals [25, 26]. In a previous study, Gullu et al showed that elevated concentrations of bilirubin may serve as a protective factor in the development of coronary flow reserve impairment, coronary microvascular dysfunction, and possibly in the development of coronary atherosclerosis. They concluded that bilirubin shows the beneficial effects independent of the known coronary risk factors [27].

Induced hyperbilirubinemia was associated with a significant improvement of endothelial function in type 2 DM [28]. Also bilirubin inhibits VCAM-1 and blocks vascular smooth muscular cell prolipheration [29].

As far as we know, there is no study available in the literature about the association between AF and serum bilirubin levels. Our study is of importance for this reason and we ascertained if there is an association between bilirubin and nonvalvular AF.

When two groups were compared in our study, serum bilirubin levels of the AF patients were significantly lower than control group. In our study, it was found that the LA diameter was associated with AF, consistent with the literature data.

We have shown for the first time that patients with nonvalvular AF have lower bilirubin levels compared to controls. Further studies are required to determine the relation between bilirubin and nonvalvular AF.

In conclusion, it was found in our study that there might be an association between nonvalvular AF and serum bilirubin. The measurement of bilirubin, also may be used to indicate increased risk of AF and AF-related adverse cardiovascular events. The most important restriction of our study is the limited number of patients. There is a need for large-scale researches about this issue.
